# A community resource for paired genomic and metabolomic data mining

**DOI:** 10.1038/s41589-020-00724-z

**Published:** 2021-02-15

**Authors:** Michelle A. Schorn, Stefan Verhoeven, Lars Ridder, Florian Huber, Deepa D. Acharya, Alexander A. Aksenov, Gajender Aleti, Jamshid Amiri Moghaddam, Allegra T. Aron, Saefuddin Aziz, Anelize Bauermeister, Katherine D. Bauman, Martin Baunach, Christine Beemelmanns, J. Michael Beman, María Victoria Berlanga-Clavero, Alex A. Blacutt, Helge B. Bode, Anne Boullie, Asker Brejnrod, Tim S. Bugni, Alexandra Calteau, Liu Cao, Víctor J. Carrión, Raquel Castelo-Branco, Shaurya Chanana, Alexander B. Chase, Marc G. Chevrette, Leticia V. Costa-Lotufo, Jason M. Crawford, Cameron R. Currie, Bart Cuypers, Tam Dang, Tristan de Rond, Alyssa M. Demko, Elke Dittmann, Chao Du, Christopher Drozd, Jean-Claude Dujardin, Rachel J. Dutton, Anna Edlund, David P. Fewer, Neha Garg, Julia M. Gauglitz, Emily C. Gentry, Lena Gerwick, Evgenia Glukhov, Harald Gross, Muriel Gugger, Dulce G. Guillén Matus, Eric J. N. Helfrich, Benjamin-Florian Hempel, Jae-Seoun Hur, Marianna Iorio, Paul R. Jensen, Kyo Bin Kang, Leonard Kaysser, Neil L. Kelleher, Chung Sub Kim, Ki Hyun Kim, Irina Koester, Gabriele M. König, Tiago Leao, Seoung Rak Lee, Yi-Yuan Lee, Xuanji Li, Jessica C. Little, Katherine N. Maloney, Daniel Männle, Christian Martin H., Andrew C. McAvoy, Willam W. Metcalf, Hosein Mohimani, Carlos Molina-Santiago, Bradley S. Moore, Michael W. Mullowney, Mitchell Muskat, Louis-Félix Nothias, Ellis C. O’Neill, Elizabeth I. Parkinson, Daniel Petras, Jörn Piel, Emily C. Pierce, Karine Pires, Raphael Reher, Diego Romero, M. Caroline Roper, Michael Rust, Hamada Saad, Carmen Saenz, Laura M. Sanchez, Søren Johannes Sørensen, Margherita Sosio, Roderich D. Süssmuth, Douglas Sweeney, Kapil Tahlan, Regan J. Thomson, Nicholas J. Tobias, Amaro E. Trindade-Silva, Gilles P. van Wezel, Mingxun Wang, Kelly C. Weldon, Fan Zhang, Nadine Ziemert, Katherine R. Duncan, Max Crüsemann, Simon Rogers, Pieter C. Dorrestein, Marnix H. Medema, Justin J. J. van der Hooft

**Affiliations:** 1 Laboratory of Microbiology, Department of Agricultural and Food Sciences, Wageningen University, Wageningen, the Netherlands; 2 Bioinformatics Group, Wageningen University, Wageningen, the Netherlands; 3 Netherlands eScience Center, Amsterdam, the Netherlands; 4 Wisconsin Institute for Discovery and Department of Plant Pathology, University of Wisconsin-Madison, Madison, WI USA; 5 Collaborative Mass Spectrometry Innovation Center, Skaggs School of Pharmacy and Pharmaceutical Sciences, University of California San Diego, La Jolla, CA USA; 6 Department of Psychiatry, University of California San Diego, San Diego, CA USA; 7 Leibniz Institute for Natural Product Research and Infection Biology e.V. Hans-Knöll-Institute (HKI), Jena, Germany; 8 Pharmaceutical Biology Department, Pharmaceutical Institute, Eberhard Karls University Tübingen, Tübingen, Germany; 9 Microbiology Department, Biology Faculty, Jenderal Soedirman University, Purwokerto, Indonesia; 10 Instituto de Ciências Biomédicas, Universidade de São Paulo, São Paulo, Brazil; 11 Center for Marine Biotechnology and Biomedicine, Scripps Institution of Oceanography, University of California San Diego, La Jolla, CA USA; 12 University of Potsdam, Institute of Biochemistry and Biology, Potsdam-Golm, Germany; 13 Department of Life and Environmental Sciences, University of California Merced, Merced, CA USA; 14 Sierra Nevada Research Institute, University of California Merced, Merced, CA USA; 15 Instituto de Hortofruticultura Subtropical y Mediterránea “La Mayora”, Universidad de Málaga-Consejo Superior de Investigaciones Científicas, Departamento de Microbiología, Universidad de Málaga, Málaga, Spain; 16 Department of Microbiology and Plant Pathology, University of California Riverside, Riverside, CA USA; 17 Molecular Biotechnology, Department of Biosciences, Goethe University Frankfurt, Frankfurt am Main, Germany; 18 Buchmann Institute for Molecular Life Sciences, Goethe University Frankfurt, Frankfurt am Main, Germany; 19 Senckenberg Gesellschaft für Naturforschung, Frankfurt am Main, Germany; 20 Max-Planck-Institute for Terrestrial Microbiology, Department of Natural Products in Organismic Interactions, Marburg, Germany; 21 Institut Pasteur, Collection of Cyanobacteria, Paris, France; 22 Pharmaceutical Sciences Division, University of Wisconsin-Madison, Madison, WI USA; 23 Laboratoire d’Analyses Bioinformatiques pour la Génomique et le Métabolisme, Génomique Métabolique, Genoscope, Institut François Jacob, CEA, CNRS, Univ Evry, Université Paris-Saclay, Evry, France; 24 Computational Biology Department, School of Computer Science, Carnegie Mellon University, Pittsburgh, PA USA; 25 Microbial Biotechnology, Institute of Biology, Leiden University, Leiden, the Netherlands; 26 Department of Microbial Ecology, Netherlands Institute of Ecology, Wageningen, the Netherlands; 27 Interdisciplinary Centre of Marine and Environmental Research), University of Porto, Porto, Portugal; 28 Faculty of Sciences, University of Porto, Porto, Portugal; 29 Department of Microbiology, University of Helsinki, Helsinki, Finland; 30 Department of Chemistry, Yale University, New Haven, CT USA; 31 Chemical Biology Institute, Yale University, West Haven, CT USA; 32 Department of Microbial Pathogenesis, Yale School of Medicine, New Haven, CT USA; 33 Department of Bacteriology, University of Wisconsin-Madison, Madison, WI USA; 34 Department of Energy Great Lakes Bioenergy Research Center, Wisconsin Energy Institute, University of Wisconsin-Madison, Madison, WI USA; 35 Adrem Data Lab, Department of Computer Science, University of Antwerp, Antwerp, Belgium; 36 Molecular Parasitology Unit, Department of Biomedical Sciences, Institute of Tropical Medicine, Antwerp, Belgium; 37 Technische Universität Berlin, Institut für Chemie, Berlin, Germany; 38 Division of Biological Sciences, University of California San Diego, La Jolla, CA USA; 39 Center for Microbiome Innovation, University of California San Diego, La Jolla, CA USA; 40 J. Craig Venter Institute, Genomic Medicine Group, La Jolla, CA USA; 41 Department of Pediatrics, School of Medicine, University of California San Diego, La Jolla, CA USA; 42 School of Chemistry and Biochemistry, Georgia Institute of Technology, Atlanta, GA USA; 43 Institute of Microbiology, Eidgenössische Technische Hochschule (ETH) Zürich, Zürich, Switzerland; 44 Department of Biological Chemistry and Molecular Pharmacology, Harvard Medical School, Harvard University, Boston, MA USA; 45 Charité, University Medicine Berlin, Berlin-Brandenburg Center for Regenerative Therapy (BCRT), Campus Virchow Klinikum, Berlin, Germany; 46 Korean Lichen Research Institute, Sunchon National University, Sunchon, Republic of Korea; 47 Naicons Srl, Milano, Italy; 48 College of Pharmacy, Sookmyung Women’s University, Seoul, Korea; 49 German Centre for Infection Research (DZIF), Tübingen, Germany; 50 Department of Chemistry, Northwestern University, Evanston, IL USA; 51 School of Pharmacy, Sungkyunkwan University, Suwon, Republic of Korea; 52 Scripps Institution of Oceanography, University of California San Diego, La Jolla, CA USA; 53 Institute for Pharmaceutical Biology, University of Bonn, Bonn, Germany; 54 Department of Chemistry, Princeton University, Princeton, NJ USA; 55 Section of Microbiology, University of Copenhagen, Copenhagen, Denmark; 56 Department of Pharmaceutical Sciences, University of Illinois at Chicago, Chicago, IL USA; 57 Department of Chemistry, Point Loma Nazarene University, San Diego, CA USA; 58 Interfaculty Institute for Microbiology and Infection Medicine Tübingen, Microbiology and Biotechnology, University of Tübingen, Tübingen, Germany; 59 Centro de Biodiversidad y Descubrimiento de Drogas, Instituto de Investigaciones Científicas y Servicios de Alta Tecnología, Panama, Republic of Panama; 60 Carl R. Woese Institute for Genomic Biology and Department of Microbiology, University of Illinois at Urbana-Champaign, Urbana, IL USA; 61 Skaggs School of Pharmacy and Pharmaceutical Sciences, University of California San Diego, La Jolla, CA USA; 62 School of Chemistry, University of Nottingham, Nottingham, UK; 63 Department of Chemistry and Department of Medicinal Chemistry and Molecular Pharmacology, Purdue University, West Lafayette, IN USA; 64 Instituto Federal de Santa Catarina, Florianópolis, Santa Catarina, Brazil; 65 Phytochemistry and Plant Systematics Department, Division of Pharmaceutical Industries, National Research Centre, Cairo, Egypt; 66 The Novo Nordisk Foundation Center for Basic Metabolic Research, Faculty of Health and Medical Sciences, University of Copenhagen, Copenhagen, Denmark; 67 Department of Biology, Memorial University of Newfoundland, St. John’s, Canada; 68 LOEWE-Centre for Translational Biodiversity Genomics, Frankfurt am Main, Germany; 69 Departamento de Fisiologia e Farmacologia, Faculdade de Medicina, Universidade Federal do Ceará, Fortaleza, Ceará Brazil; 70 University of Strathclyde, Strathclyde Institute of Pharmacy and Biomedical Sciences, Glasgow, UK; 71 School of Computing Science, University of Glasgow, Glasgow, UK; 72 Department of Pharmacology and Pediatrics, University of California San Diego, La Jolla, CA USA

**Keywords:** Computational biology and bioinformatics, Databases, Systems biology, Metabolomics, DNA

## Abstract

Genomics and metabolomics are widely used to explore specialized metabolite diversity. The Paired Omics Data Platform is a community initiative to systematically document links between metabolome and (meta)genome data, aiding identification of natural product biosynthetic origins and metabolite structures.

Interactions between bacteria, fungi, plants, and animals, as well as their environments are often facilitated through specialized metabolites, also known as natural products. These specialized metabolites are molecules naturally produced by organisms that are not strictly required for survival but may confer an advantage to the producing organism, such as the inhibition of nearby species competing for nutritional resources. The chemical structures and functions, as well as the biosynthetic origins of such metabolites, are largely hidden, especially in complex environments. To understand and harness these chemical interactions, it is crucial to study their genetic and structural bases. However, the confident recognition, dereplication, and prioritization of specialized metabolites in complex mixtures remains very challenging. While individual efforts to interpret the chemical and genetic languages have been largely successful in connecting genes and molecules^1,2^, large-scale correlations leveraging complementary chemical and genomic data have yet to be realized.

The research community has generated a wealth of genomic and metabolomic data, which has been deposited in dedicated repositories, and tools for mining these data separately are being developed rapidly. Platforms such as the antibiotics and Secondary Metabolite Analysis Shell (antiSMASH)^3^ and PRediction Informatics for Secondary Metabolomes (PRISM)^4^ use genomic information to annotate biosynthetic gene clusters (BGCs), a set of genes that encode the producing framework for metabolites of diverse chemical classes, such as polyketides, peptides and terpenoids. The antiSMASH database and the Joint Genome Institute’s (JGI’s) Integrated Microbial Genomes and Microbiomes (IMG/M)/Atlas of Biosynthetic Gene Clusters (IMG/ABC) database^5^ contain tens of thousands of BGCs identified in publically available genomes, while the Minimum Information about a Biosynthetic Gene cluster (MIBiG)^6^ database connects over 2,000 BGCs to the specialized metabolites for which they encode the biosynthetic pathways. On the metabolomics side, mass spectrometry (MS) has become the most commonly used technique for performing high-throughput measurements^2^. Data repositories and analysis platforms such as the Global Natural Product Social Molecular Networking-Mass Spectrometry Interactive Virtual Environment (GNPS-MassIVE)^7^, MetaboLights^8^, and the Metabolomics Workbench^9^ facilitate the sharing, processing, and analysis of MS data. These platforms, along with spectral libraries^2^, such as the GNPS spectral library, METLIN, MassBank, and the commercially available NIST library, provide resources for reference mass spectra of a wide range of chemical structures, thereby aiding metabolite annotation. Together, these resources provide the basis for sharing and reusing genomic and metabolomic data and structural annotations and have spurred the development of numerous algorithms for mining these information-dense data.

Several studies and tools have started to explore the combination of genomic and metabolomic data to enhance metabolite annotation, dereplication, and prioritization workflows. While MS-based metabolomics provides increasing amounts of information related to the metabolite structures present in complex mixtures, it faces inherent limitations with respect to structural identification. To address this, several tools, such as GNPS-based molecular networking^7^ and mass spectrometry to latent dirichlet allocation (MS2LDA) substructure discovery^10^, have been proposed that computationally exploit tandem mass spectrometry (MS/MS) fragmentation spectra to map relationships between metabolites in networks and identify (shared) substructures, thereby facilitating metabolite annotation. Genomics has also been used to provide complementary structural information through the biotransformations encoded in biosynthetic machinery^1^, as well as a way to link specialized metabolites to their producers via BGCs that are mined from genome sequences from known organisms. Integrative strategies have been described for bacterial^11^, fungal^12^, and plant^13^ specialized metabolites. A series of tools and approaches, mostly targeting biosynthetically modular natural products such as peptides and glycosides, have been introduced over the last decade to integrate genome and metabolome data, such as peptidogenomics^11^, MetaMiner^14^, GRAPE-GARLIC^15^ and metabologenomics^16^. These tools show the potential of combined omics approaches to accelerate natural product discovery.

It has become standard procedure to deposit genomic information to public databases, such as the National Center for Biotechnology Information’s (NCBI’s) GenBank^17^ or JGI’s IMG/M^5^, and it is becoming increasingly common to submit mass spectrometry data to repositories such as GNPS-MassIVE^7^, MetaboLights^8^ or Metabolomics workbench^9^. However, there is currently no straightforward way to connect different types of omics data that are derived from the same biological source. It often takes extensive literature review to determine which omics data belong to the same species, organism, or sample, and therefore constitute ‘paired’ datasets, making reuse of these data challenging and time consuming. Additionally, there is no straightforward way to obtain consistent metadata for such links. To facilitate large-scale, effective integration of these data, it is vital to have a community-driven online resource that stores annotated links between paired datasets. Here, we refer to paired data as genomic data (specifically a genome or metagenome assembly) and metabolomic data (specifically MS/MS data) that originate from the same source. So far, no such platform supporting natural product discovery has been available. The value of integrating different data types and organizing sample metadata is increasingly recognized by the scientific community. For example, the BioStudies^18^ and BioSample^19^ databases facilitate the capture and organization of various omics data types and sample information. In particular, the BioStudies database supports linkage between genomics and metabolomics studies; however, links between genome-mining resources, such as MIBiG, and natural product metabolomics platforms, such as GNPS-MassIVE, are currently not documented in this database.

Here we introduce the Paired Omics Data Platform (PoDP) to streamline access to paired omics data so that both humans and computers can access and read paired datasets and can also record and exploit validated links between BGCs and metabolites (https://pairedomicsdata.bioinformatics.nl/). In addition to linking these omics data types, the platform stores essential metadata (i.e., growth media, extraction solvent, and ionization mode) using existing ontology where available, thus facilitating reuse of for-the-user relevant sections of paired data. This platform will boost the successful integration of unsupervised data-mining strategies to fine-tune the structural annotation of modular natural product classes and include yet-unknown classes of natural products. This will aid in structural and functional annotations of natural products and the genes responsible for their production, and we anticipate that this will help uncover the potential producers of molecules in nature. Finally, registering these links in a standardized way gives the community an invaluable resource of Findable, Accessible, Interoperable, and Reusable (FAIR)^20^ data.

## Standards for paired data

**Fig. 1:**
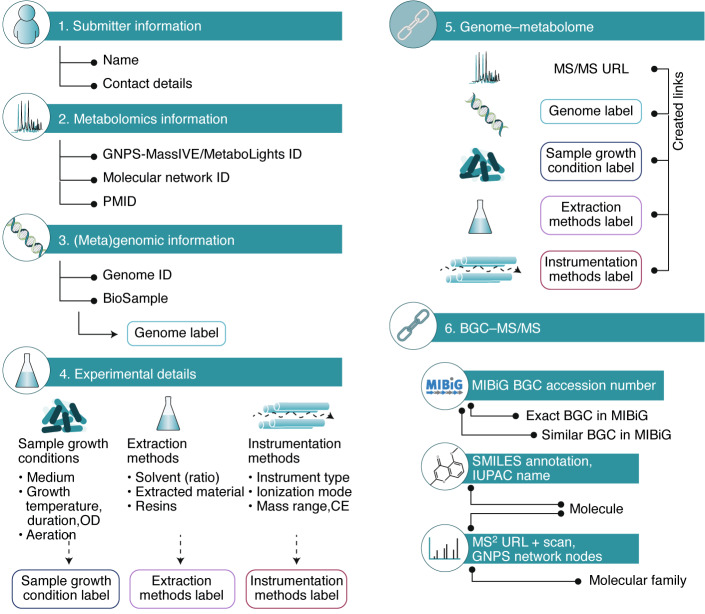
Overview of the Paired Omics Data Platform. The PoDP links genomic and metabolomic data deposited in public repositories, accompanied by minimal metadata. The platform documents basic submitter information as well as accession numbers of the metabolome and genome data. Standardized logging of key experimental details enables users to better search and compare datasets, and user-defined labels of genome and experimental details (sections 3 and 4) enable straightforward submission of multiple links. The core of the platform consists of links between the genomic and metabolomic datasets and links between BGCs and MS/MS spectra, which facilitate data integration. PMID, PubMed ID; OD, optical density; CE, collision energy; SMILES, simplified molecular-input line-entry; IUPAC, International Union of Pure and Applied Chemistry; MS^2^, MS/MS fragmentation.

The aim of the PoDP is to connect public metabolomics datasets to their genomic origins. The PoDP does not store any metabolomics or genomics datasets, but captures metadata defining pairs of omics datasets in existing public databases and platforms already validated and utilized by the genomics and metabolomics communities. The PoDP consists of a six-section form for easy and quick input of data (Fig. 1). The metadata is organized in projects that can consist of multiple related experiments, identified by their MassIVE accession or MetaboLights study identifier. The (meta)genomes(s) used in these experiments can all be added to the same project via a public database identifier (e.g., a NCBI GenBank accession number or JGI Genome ID), with the user creating easy-to-recall genome labels for each (meta)genome. Minimal metadata with information about sample preparation and data collection are recorded in a modular way, allowing for multiple experimental set-ups within one project. Furthermore, through BioSample accession IDs, metadata stored elsewhere can be linked to (meta)genome(s) as well. User-specified metadata labels are also used for easy recall in the linking step, in which a URL for a specific set of MS spectra is linked with the genome label and metadata labels to create a genome–metabolome link. To create a BGC–MS/MS link, a MIBiG identifier for the same or similar BGC can be linked with a MS/MS URL and scan number of a single measured molecule or molecular network nodes (representing unique measured molecules) in a molecular family (a group of structurally related molecules identified by similar fragmentation patterns). This approach thus stimulates the submission of validated gene clusters to the MIBiG repository in order to make a BGC–MS/MS link in the PoDP.

By obtaining iterative feedback from a group of early users from various research groups, we narrowed down the required metadata in the PoDP to the minimum information needed to make meaningful links between genomic and metabolomic data relevant to the community. Capturing the full range of relevant variables in any given experiment in a standardized and machine-readable format would lead to a very complex and tedious data entry process. Therefore, a balance was struck between flexible and user-friendly data entry, maintaining machine readability for future large-scale analyses. By standardizing and connecting to ontologies only the most relevant information that could substantially affect the metabolites produced, extracted, and detected by MS, we arrived at a set of minimal metadata required for submission.

To enable machine readability of the data, ontologies are used to standardize response options wherever possible. This ensures that a global community can use the same term for a given piece of metadata and use these ontologies to make accurate and meaningful selections of data to analyze. For example, researchers can reliably select and obtain only datasets that use tryptic soy broth for culture or only metagenomic datasets derived from aquatic invertebrates, or just the fraction of paired datasets in which the MS data was obtained in positive ionization mode. For metadata categories with numerous options, in which all possibilities cannot be captured by standard ontologies, an “Other” category is provided for further explanation. Free text entered in the “Other” boxes is inherently not machine-readable but gives an option for customization by the user and can help to keep important but non-standardized records of the paired data. Furthermore, all fields including the “Other” boxes can be searched to find projects containing specific data.

## Preliminary dataset statistics

An initial call to deposit paired datasets in the PoDP was met with enthusiasm from the research community. Over 45 laboratories from 10 countries have contributed 70 paired datasets. Those 70 projects (Box 1) contain 4,853 MS samples associated with sequenced source material. Of the more than 2,600 different genomic sources deposited, 1,306 are metagenomes, 1,268 are genomes, and 42 are metagenome-assembled genomes. The impressive collection of over 4,800 genome–metabolome links is accompanied by metadata: 155 sample preparation methods, 100 extraction methods, and 75 instrumentation methods. Furthermore, 114 links between BGCs and their associated MS/MS spectra are registered in the platform. These community-curated data are regularly archived to a Zenodo dataset and made available for download in JSON format.

The PoDP encourages adherence to FAIR principles^20^, requiring data to already be deposited in databases and made publicly available before being entered in the PoDP. Presence of a project in the PoDP will increase the findability of those data, results, and publications, while allowing researchers to perform new analyses on existing publicly available data without the need to generate new data. As part of this community effort, a number of projects deposited in the PoDP made their data publicly available to allow submission into the platform; thus far, over 680 metabolomics samples and over 70 genomic sources, including five BGCs newly uploaded to MIBiG, were made public. For example, the PoDP stimulated the upload of metabolomics data to MassIVE for a collection of 120 sequenced *Streptomyces* strains for which genomics data was previously published^21^. In another example, 20 metagenomes from marine sediments were made public for the platform. Additionally, some datasets were acquired and made publicly available expressly for deposition into the PoDP. In one case, a research group with 44 already sequenced cyanobacterial strains^22^ was inspired to acquire metabolomics data for each strain so that the paired data could be uploaded to the PoDP.

To better view the data encompassed by the PoDP, users can search for projects under the “List” tab, using keywords to find studies of interest. For example, to find paired data resulting from a *Streptomyces* or *Salinispora* species, searching for the genera (“*Streptomyces* | *Salinispora*”) will result in the projects (currently 18) that measured *Streptomyces* or *Salinispora* strains. Likewise, to compare projects that used methanol to extract cell pellets, searching “methanol + cells” retrieves projects that used methanol to extract cell pellets. To obtain more detail on the metadata contained in each project, users can navigate to the project page by clicking on the project identifier. There, users can find details about the genome or metagenome when clicking on the label, which will then provide a link to the publically available genomic data. Likewise, the publically available MS data can be downloaded directly from the link provided. Clicking on the Sample Growth, Extraction, and Instrumentation Methods labels will display the corresponding metadata.

Box 1 Preliminary submissions to the PoDP

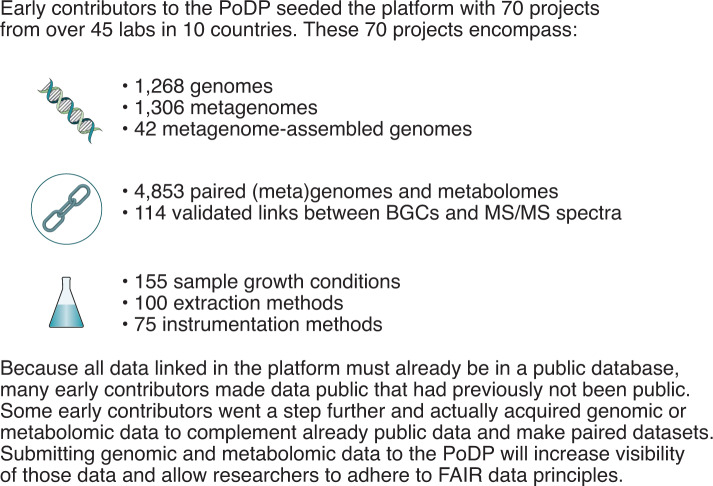

## Applications of the platform

**Fig. 2:**
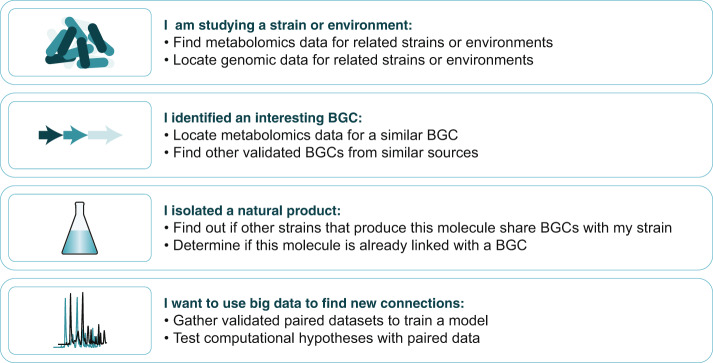
Example use cases of the Paired Omics Data Platform. Users may approach the PoDP using genomic or metabolomic data (or using metadata) and exploit the links provided to generate new hypotheses about their primary data. Specifically, genomic data may enable new hypotheses about the structures or biosynthetic pathways for an identified molecule or mass feature, while metabolomic data may provide new hypotheses regarding the product(s) of a BGC. Integrative computational approaches allow scaling these analyses to systematic and comprehensive efforts.

The PoDP can be used in both basic and advanced ways. In a basic way, researchers from across disciplines can apply linked data for numerous applications (Fig. 2). With linked data, we refer to a BGC that can be experimentally linked to a MS/MS spectrum or a molecular family. For example, a natural product chemist who isolates a molecule from a cyanobacterium can use the PoDP to find mass spectra from genetically similar cyanobacteria for comparative metabolomics analyses. A biologist who has identified a BGC of interest and has MS data for the producing strain can download data for the products of similar BGCs and their products to determine whether the BGC is novel and/or to guide molecule isolation. Scientists from all fields can find reliable paired data for use in their own research while also contributing their data for future community use. The importance of consistent metadata cannot be underestimated, and we welcome the development of curated resources such as the Natural Products Atlas^23^ that aim to create coherent records for microbial natural products. Combined with the PoDP, this gives researchers complementary resources to mine for natural product structures, their producers, and available omics data.

Furthermore, more advanced applications are possible utilizing large-scale computational approaches (Fig. 2). Several algorithmic strategies to link genomics and metabolomics data to chart specialized metabolic diversity have been suggested, including correlation- and feature-based matching^2^. Both types of linking benefit from systematically curated datasets of related organisms with BGCs and metabolites occurring in various samples. With the PoDP in place now, these strategies can be used more effectively to select appropriate datasets to start mining for novel links. Moreover, algorithms to score and rank links between BGCs and metabolites are easier to develop and benchmark: for example, a new set of scores was recently proposed using a number of PoDP datasets with validated BGC–metabolite links to demonstrate the effect of the novel scoring system within the newly introduced NPLinker framework^24^.

## Moving forward with FAIR data

The amount of preliminary data deposited and the enthusiasm from the community for the PoDP reaffirm the need for such a repository of paired public datasets. Feedback from early users also indicated an eagerness to include additional kinds of data in the future. Presently, the PoDP is expressly for linking MS/MS data and whole-genome or metagenome data. Potentially, the PoDP could be developed to include other types of spectral data, like full scan (MS^1^) metabolomics mass spectrometry data and NMR, as well as proteomics data. Additionally, different kinds of genomic data could be facilitated, including 16S rRNA or other amplicon sequences, transcriptomic data, and genetic manipulation or heterologous expression data. Such additions will further fuel integrated omics analysis tools and approaches, a field that has gained much traction recently^25^.

The PoDP requires researchers to deposit their data in public databases, stimulating the upload of data by early users, which is exemplified by more than 1,800 GNPS-MassIVE and MetaboLights submissions just prior to submitting these data in the PoDP. As a FAIR data platform, the PoDP not only facilitates reuse of data, but also promotes the work of researchers who submit their data to the PoDP, through increased publication visibility. Future efforts to (re)use these data by connecting to other platforms and programs for analyzing paired data, such as NPLinker^24^, will further the field of natural product prediction and discovery.

### Reporting Summary

Further information on research design is available in the Nature Research Reporting Summary linked to this article.

## Supplementary information

Reporting Summary

## Data Availability

Each project can be downloaded from the website individually as a JSON file. The (meta)genome and metabolome datasets can be found in their public repositories. All PoDP projects are archived monthly to Zenodo at 10.5281/zenodo.3736430.

## Code availability

The software is licensed under the Apache 2.0 open source license and the source code can be found on GitHub (https://github.com/iomega/paired-data-form), which includes the dependencies of the software. Each software release is archived to Zenodo at 10.5281/zenodo.2656630. A full description of how the platform was built can be found on https://pairedomicsdata.bioinformatics.nl/methods.
