# Supplementary material for: A community resource for paired genomic and metabolomic data mining
Source: Nat Chem Biol. 2021 Feb 15;17(4):363–8. doi: 10.1038/s41589-020-00724-z (PMC7987574; doi:10.1038/s41589-020-00724-z)
Supplement: Supplementary file 1 — Reporting Summary [file 41589_2020_724_MOESM1_ESM.pdf]

nature research

Corresponding author(s): Justin J.J. van der Hooft

Last updated by author(s): Sep 9, 2020

Reporting Summary

Nature Research wishes to improve the reproducibility of the work that we publish. This form provides structure for consistency and transparency in reporting. For further information on Nature Research policies, see Authors & Referees and the Editorial Policy Checklist.

Statistics

For all statistical analyses, confirm that the following items are present in the figure legend, table legend, main text, or Methods section.

n/a	Confirmed	
☒	☐	The exact sample size (*n*) for each experimental group/condition, given as a discrete number and unit of measurement
☒	☐	A statement on whether measurements were taken from distinct samples or whether the same sample was measured repeatedly
☒	☐	The statistical test(s) used AND whether they are one- or two-sided<br>*Only common tests should be described solely by name; describe more complex techniques in the Methods section.*
☒	☐	A description of all covariates tested
☒	☐	A description of any assumptions or corrections, such as tests of normality and adjustment for multiple comparisons
☒	☐	A full description of the statistical parameters including central tendency (e.g. means) or other basic estimates (e.g. regression coefficient) AND variation (e.g. standard deviation) or associated estimates of uncertainty (e.g. confidence intervals)
☒	☐	For null hypothesis testing, the test statistic (e.g. *F*, *t*, *r*) with confidence intervals, effect sizes, degrees of freedom and *P* value noted<br>*Give P values as exact values whenever suitable.*
☒	☐	For Bayesian analysis, information on the choice of priors and Markov chain Monte Carlo settings
☒	☐	For hierarchical and complex designs, identification of the appropriate level for tests and full reporting of outcomes
☒	☐	Estimates of effect sizes (e.g. Cohen's *d*, Pearson's *r*), indicating how they were calculated

Our web collection on statistics for biologists contains articles on many of the points above.

Software and code

Policy information about availability of computer code

Data collection	The Paired Omics Data Platform is running at https://pairedomicsdata.bioinformatics.nl/.<br>The software is licensed under the Apache 2.0 open source licence and the source code can be found on GitHub (https://github.com/iomega/paired-data-form), which includes the dependencies of the software. Each software release is archived to Zenodo with https://doi.org/10.5281/zenodo.2656630 as DOI. Installation guide available at https://github.com/iomega/paired-data-form/blob/master/README.md . A demo dataset can be loaded from the project submission form.<br>The current Paired Omics Data Platform was built using the following software: React (v16.13.1). The platform runs using Docker Compose (v1.25.4) with containers for the web application, web service and redis queue. The web service has an OpenAPI (v3.0.3) specification (https://www.openapis.org/) which can be used to submit and retrieve projects in a programmatic manner. Additional information on the genomes entered into the platform was retrieved from GenBank using the public genome identifiers in a project. The Paired Omics Data Platform offers textual searches using elastic search (v7.6.2).
Data analysis	Acces to the data and data analysis is done through the same software as data collection, so https://github.com/iomega/paired-data-form and https://doi.org/10.5281/zenodo.2656630

For manuscripts utilizing custom algorithms or software that are central to the research but not yet described in published literature, software must be made available to editors/reviewers. We strongly encourage code deposition in a community repository (e.g. GitHub). See the Nature Research guidelines for submitting code & software for further information.

Data

Policy information about availability of data

All manuscripts must include a data availability statement. This statement should provide the following information, where applicable:

- Accession codes, unique identifiers, or web links for publicly available datasets
- A list of figures that have associated raw data
- A description of any restrictions on data availability

The Paired Omics Data Platform is running at https://pairedomicsdata.bioinformatics.nl/.
Each project can be downloaded from the website individually as a JSON file. The publicly available (meta)genome and metabolome datasets can be found in their public repositories (NCBI GenBank, RefSeq, JGI IMG, ENA, MGnify, MassIVE, MetaboLights). All PoDP projects are archived monthly to Zenodo with https://doi.org/10.5281/zenodo.3736430 as DOI.
There are no restrictions to access any of the data and we put in a large effort to make the Paired Omics Data Platform adhere to the FAIR data principles.

Field-specific reporting

Please select the one below that is the best fit for your research. If you are not sure, read the appropriate sections before making your selection.

☒ Life sciences ☐ Behavioural & social sciences ☐ Ecological, evolutionary & environmental sciences

For a reference copy of the document with all sections, see nature.com/documents/nr-reporting-summary-flat.pdf

Life sciences study design

All studies must disclose on these points even when the disclosure is negative.

Sample size	No sample size was precalculated; rather, as many as possible of the publicly available genomic and metabolomic data was used for the data linking, thus maximizing the amount of current input data into the Paired Omics Data Platform.
Data exclusions	No data was excluded.
Replication	In our study, no statistical analyses were done, so no replication was needed. However, the Paired Omics Data Platform does allow to take up replicate mass spectrometry measurements to facilitate statistical analyses of data from the recorded links.
Randomization	Randomization was not relevant for our study, as we did not perform group comparisons.
Blinding	No blinding was performed in our study as no group comparisons were performed.

Reporting for specific materials, systems and methods

We require information from authors about some types of materials, experimental systems and methods used in many studies. Here, indicate whether each material, system or method listed is relevant to your study. If you are not sure if a list item applies to your research, read the appropriate section before selecting a response.

Materials & experimental systems

n/a	Involved in the study
☒	☐ Antibodies
☒	☐ Eukaryotic cell lines
☒	☐ Palaeontology
☒	☐ Animals and other organisms
☒	☐ Human research participants
☒	☐ Clinical data

Methods

n/a	Involved in the study
☒	☐ ChIP-seq
☒	☐ Flow cytometry
☒	☐ MRI-based neuroimaging
